# Differences in the Cancer Burden and Current Funding of NCI-Designated Cancer Centers

**DOI:** 10.1001/jamanetworkopen.2025.24564

**Published:** 2025-08-01

**Authors:** Todd Burus, Caree R. McAfee, Pamela C. Hull

**Affiliations:** 1 Markey Cancer Center, University of Kentucky, Lexington; 2 Department of Behavioral Science, College of Medicine, University of Kentucky, Lexington

## Abstract

**Importance:**

National Cancer Institute (NCI)-designated clinical and comprehensive cancer centers are required to define a geographic catchment area (CA) in which they focus research, outreach, and engagement efforts for the purposes of reducing cancer burden, particularly among certain vulnerable populations. A comprehensive analysis of the cancer burden of these catchment areas and their association with cancer center funding through the Cancer Center Support Grant (CCSG) has not been performed.

**Objective:**

To examine CA cancer burden indicators and cancer center CCSG funding during the 2023 federal fiscal year (FY23).

**Design, Setting, and Participants:**

This population-based cross-sectional study assessed cancer center cancer rates, sociodemographic variables, and reported CCSG funding. Participants included NCI-Designated Clinical and Comprehensive Cancer Centers as of FY23. Data were analyzed from August to December 2024.

**Exposures:**

Race and ethnicity, socioeconomic status, disability status, urbanicity.

**Main Outcomes and Measures:**

Main outcomes included CA cancer rates with 95% CI and various population percentage estimates, as well as cancer center CCSG direct costs for FY23. Adjusted CCSG funding by CA population size was also assessed. The association between cancer burden indicators and population-adjusted CCSG funding was estimated using Spearman rank correlation.

**Results:**

This study included 64 NCI-Designated Cancer Centers (11 clinical [17.2%], 53 comprehensive [82.8%]) with CAs covering 297 040 253 people. Individual CA populations ranged from 1.4 million to 29.9 million people, with substantial variation in sociodemographic compositions. CA cancer incidence ranged from 369.2 (95% CI, 367.5 to 370.8) to 518.7 (95% CI, 514.2 to 523.2) cases per 100 000 persons. Cancer mortality ranged from 115.2 (95% CI, 114.3 to 116.1) to 181.1 (95% CI, 179.5 to 182.7) deaths per 100 000 persons. Population-adjusted CCSG funding showed no association with cancer rates, but was positively associated with rural populations (*r* = 0.356; 95% CI, 0.119 to 0.555) and persons living with a disability (*r* = 0.378; 95% CI, 0.143 to 0.572) and negatively associated with populations belonging to a racial or ethnic minority group (*r* = −0.354; 95% CI, −0.553 to −0.116).

**Conclusions and Relevance:**

In this cross-sectional study of NCI-Designated Cancer Centers, there were substantial disparities in cancer burden across catchment areas. CCSG funding did not align well with this distribution, raising questions about whether funding allocations effectively support cancer centers serving high-need CAs. In addition to existing criteria, consideration of CA cancer burden and vulnerable populations in determining CCSG funding amounts could enhance efforts to reduce cancer burden and disparities.

## Key Points

**Question:**

Is there an association between Cancer Center Support Grant (CCSG) funding of National Cancer Institute (NCI)-Designated Cancer Centers and the cancer burden of their catchment areas?

**Findings:**

This population-based cross-sectional study found no association between CCSG funding and catchment area cancer incidence or mortality rates. Funding was positively associated with catchment area percentages of rural population and persons living with a disability and negatively associated with the percentage of population belonging to a racial or ethnic minority group.

**Meaning:**

These findings suggest that CCSG funding of NCI-Designated Cancer Centers does not currently align well with the distribution of cancer-related needs across catchment areas.

## Teaser

This cross-sectional study examines the association between Cancer Center Support Grant funding of National Cancer Institute–Designated Cancer Centers and the cancer burden of their catchment areas.

## Introduction

The US Congress defined the modern structure of the National Cancer Institute (NCI) through passage of the National Cancer Act of 1971.^1^ Among other innovations, this act established the NCI’s cancer centers program—a strategic initiative to recognize institutions that do exemplary research in the prevention, diagnosis, and treatment of cancer. As of May 2025, there were 73 NCI-designated basic laboratory, clinical, or comprehensive cancer centers across the US.^2^ These centers receive funding from the NCI through the P30 Cancer Center Support Grant (CCSG) funding mechanism to support infrastructure for conducting research with national and global reach. In addition, the CCSG funding announcement was updated during the 2010s to include a mandate that all clinical and comprehensive cancer centers define a population-based and geographically defined catchment area. The expectation of this mandate is that a cancer center will focus research, outreach, and engagement activities toward their catchment area with the goal of contributing to the reduction of its overall cancer burden.^3,4,5^

Given the elevated importance of catchment areas in the cancer centers program, 2 previous studies have provided overviews of their composition. In 2022, Delnero et al^6^ published a report on the catchment areas of NCI-designated cancer centers as of 2019, including total population, certain demographics (sex, age, and race or ethnicity), and cancer mortality. They found great diversity in the geographic scope of defined catchment areas, with some smaller than a single county while others covered parts or all of multiple states. This study resulted in an interactive dashboard that continues to be maintained on the NCI website.^8^ Also in 2022, Leader et al^7^ reported on the US counties covered in the catchment areas of 89 American Association of Cancer Institute members, which included all NCI-designated cancer centers, along with overlays for county-level cancer incidence, cancer mortality, and population density. While these articles helped to establish awareness about the wide variety of cancer center catchment areas, to our knowledge, no previous study has reported on cancer incidence rates calculated for each catchment area as a single geographic unit. Moreover, neither study looked at how, if at all, the composition and cancer burden of catchment areas relate to funding cancer centers receive through the CCSG—a question of importance given that catchment areas have been a requirement of the funding announcement for over a decade. In this study, we examined the updated composition and cancer burden of NCI-designated cancer center catchment areas—including a novel analysis of catchment area cancer incidence rates—and to what extent the dollar amount of awards under the P30 CCSG funding mechanism was associated with existing catchment area population cancer needs.

## Methods

This study did not include human participant data and, thus, did not require institutional review board approval or patient consent, in accordance with the Common Rule. We followed the Strengthening the Reporting of Observational Studies in Epidemiology (STROBE) reporting guideline for cross-sectional studies.

### Data Sources

A list of NCI-designated clinical and comprehensive cancer centers and their respective catchment areas during 2023 was obtained from the NCI website.^8^ We used county-level catchment area approximations for this study to aid in statistical analysis. As such, subcounty areas included in 2 catchment areas were expanded to the entire county containing it. The catchment area for the University of Hawaii Cancer Center was reduced to only include the state of Hawaii due to data availability, excluding the US affiliated Pacific Islands. Eight counties were added to the catchment area of Moffitt Cancer Center due to a known error on the NCI website.

Catchment area demographics were calculated using the US Census Bureau’s American Community Survey (ACS) 2019-2023 5-year Estimates.^9^ We included total population and National Institutes of Health–designated populations that experience health disparities as examples of vulnerable groups: people from racially or ethnically minoritized groups, people living below poverty, people living in rural areas, and people living with disabilities. Sexual and gender minority groups were not analyzed due to the lack of an appropriate variable in the ACS. Racial or ethnic minority groups defined by the Census Bureau included Hispanic (any race), non-Hispanic American Indian or Alaska Native, non-Hispanic Asian or Pacific Islander, non-Hispanic Black, and other non-Hispanic race. Other non-Hispanic races included unknown non-Hispanic race and 2 or more non-Hispanic races. Respondents self-reported race and ethnicity according to available categories. Rural population was based on counties with a 2013 Rural Urban Continuum Code (RUCC) of 4-9.^10^ Disability was determined according to self-reported conditions impairing or serious difficulty with hearing, vision, cognition, ambulation, self-care, or independent living.^11^

Catchment area-level cancer incidence was obtained using the US Center for Disease Control and Prevention’s (CDC) US Cancer Statistics (USCS) Incidence Analytic Database, 2023 Submission.^12^ Catchment area-level cancer mortality was obtained using the CDC’s National Center for Health Statistics county-level underlying mortality for 1990 to 2021. Incidence and mortality data were collected for all cancer sites combined and 4 individual cancer sites with grade A or B recommended screenings from the US Preventive Services Task Force (female breast, lung, colon and rectum, and cervix).^13,14,15,16^ Catchment area incidence data were not collected for Indiana University Melvin and Bren Simon Comprehensive Cancer Center or University of Chicago Comprehensive Cancer Center due to data in Indiana not meeting USCS publication criteria during the years considered. Additional data were collected by patient race, ethnicity, and urbanicity. The CDC used race and ethnicity data extracted from medical records to create the following minority racial and ethnic categories: Hispanic (all races), non-Hispanic American Indian or Alaska Native, non-Hispanic Asian or Pacific Islander, non-Hispanic Black, and non-Hispanic unknown race. Rural populations were determined by county of residence having RUCC 4-9. Cancer incidence and mortality rates were not available for any of the other health disparities populations.

P30 CCSG total and direct cost funding amounts were obtained from the National Institutes of Health RePORTER application programming interface using the R package ‘reporter.nih’ (R Project for Statistical Computing).^17^ CCSG funding amounts for the 2023 fiscal year (FY23) were obtained by including all applicable P30 Center Core Grants (opportunity numbers PAR-17-095, PAR-20-043, and/or PAR-21-321) and administrative supplements (opportunity number PA-20-272).

### Statistical Analysis

Cancer incidence and mortality rates with 95% CI were calculated for 2017 to 2021 and age-adjusted according to the 2000 US standard population in SEER*Stat version 8.4.3 (NCI). No rates required suppression (ie, less than 16 cases or less than 10 deaths). Associations between cancer burden indicators and CCSG funding were calculated using Spearman rank correlation to account for lack of linearity. All significance tests were 2-sided and assessed using *P* < .05. All analyses were performed in the R statistical programming language version 4.3.3 (R Project for Statistical Computing). Data were analyzed from August to December 2024.

## Results

### Catchment Area Characteristics

**Figure 1.**
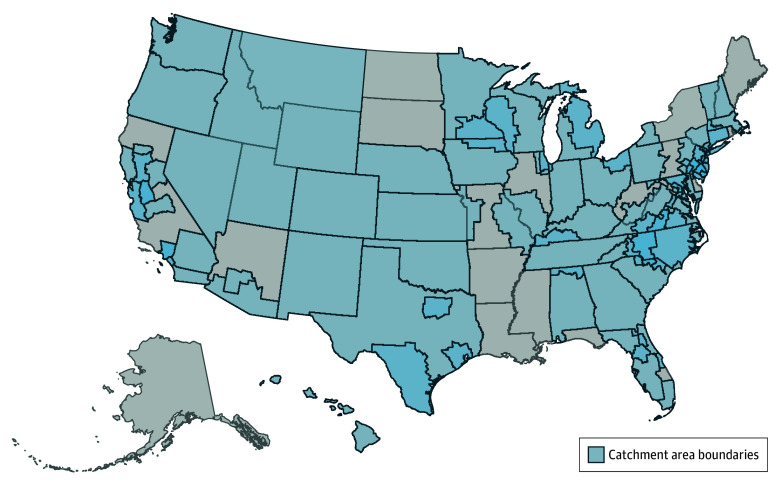
Cancer Center Catchment Areas Map of county-level catchment areas for the 64 National Cancer Institute-Designated Cancer Centers required to have a population-based and geographically defined catchment area in 2023.

During 2023, there were 64 NCI-designated cancer centers (11 clinical [17.2%], 53 comprehensive [82.8%]) with self-defined catchment areas, covering a combined 297 040 253 of the 332 387 540 people living in the US (89.4%) (Figure 1 and eTable 1 in Supplement 1). Catchment area compositions varied widely. Individual catchment area populations ranged from 1 419 250 persons at Montefiore Einstein Comprehensive Cancer Center to 29 919 552 persons at Memorial Sloan-Kettering Cancer Center, with a median (IQR) population size of 6 170 738 (4 100 622-9 412 362). Racial or ethnic minority groups as a percentage of catchment area population ranged from 233 883 of 2 033 088 (11.5%) at Dartmouth Cancer Center to 1 294 134 of 1 419 250 (91.2%) at Montefiore Einstein Comprehensive Cancer Center. Eighteen cancer centers had no rural population, while Dartmouth Cancer Center had the largest rural population (932 847 of 2 033 088 [45.9%]). Percentage of catchment area population living below poverty ranged from 160 359 of 1 970 341 (8.1%) for Dartmouth Cancer Center to 374 138 of 1 388 414 (26.9%) for Montefiore Einstein Comprehensive Cancer Center. The percentage of catchment area population living with a disability ranged from 570 696 of 6 379 236 (8.9%) for Georgetown Lombardi Comprehensive Cancer Center to 784 920 of 4 429 333 (17.7%) for the University of Kentucky Markey Cancer Center. Individual catchment areas covered geographic regions between 57 square miles for Montefiore Einstein Comprehensive Cancer Center and 523 890 square miles for Huntsman Cancer Institute. Nationally, all or part of 42 states and Washington DC were included in at least 1 catchment area, and 2495 of 3144 counties.

### Cancer Incidence and Mortality by Catchment Area

**Figure 2.**
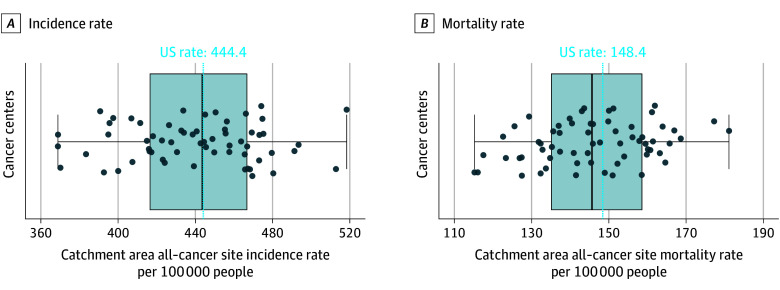
Distributions of Catchment Area All–Cancer Site Incidence and Mortality Rates, 2017-2021 A) Catchment area all–cancer site incidence rates per 100 000 people (n=62), (B) catchment area all–cancer site mortality rates per 100 000 people (n=64). Catchment area incidence rates were not calculated for Indiana University Melvin and Bren Simon Comprehensive Cancer Center and University of Chicago Comprehensive Cancer Center due to 2017 to 2021 county-level cancer incidence rates in Indiana not meeting USCS publication criteria. Boxplot represents 25th percentile, 50th percentile, and 75th percentile. Whiskers extend to 1.5 times the IQR. Dots indicate individual cancer centers. Blue vertical lines indicate US rates.

In 2017 to 2021, the US had an age-adjusted all–cancer site incidence rate of 444.4 cases per 100 000 population (95% CI, 444.1-444.7). Catchment area all–cancer site incidence rates ranged from 369.2 (95% CI, 367.5-370.8) for the UCLA Health Jonsson Comprehensive Cancer Center and USC Norris Comprehensive Cancer Center to 518.7 (95% CI, 514.2-523.2) for the Roswell Park Comprehensive Cancer Center (Figure 2A and eTable 2 in Supplement 1).

The 3 highest catchment area racial or ethnic minority group cancer incidence rates were observed for the University of Oklahoma Health Stephenson Cancer Center (499.6; 95% CI, 492.9-506.5), Roswell Park Comprehensive Cancer Center (494.8; 95% CI, 482.2-507.7), and the University of Wisconsin Carbone Cancer Center (487.8; 95% CI, 479.9-495.8) (eTable 3 in Supplement 1). USC Norris and UCLA Health Jonsson (331.6; 95% CI, 329.6-333.5) and City of Hope Comprehensive Cancer Center (339.9; 95% CI, 338.3-341.4) had the lowest catchment area incidence rates among racial or ethnic minority groups. Breakdowns of incidence rates by specific racial or ethnic minority groups are available in eTables 4 to 7 in Supplement 1. The highest catchment area rural population cancer incidence rates were observed for Markey Cancer Center (522.4; 95% CI, 518.2-526.6), UF Health Cancer Center (514.8; 95% CI, 505.6-524.3), and Roswell Park Comprehensive Cancer Center (504.3; 95% CI, 495.0-513.7) (eTable 8 in Supplement 1).

Among the four screening-detected cancers, Markey Cancer Center had the highest catchment area incidence rates of lung cancer (84.4; 95% CI, 83.3-85.5) and colorectal cancer (45.9; 95% CI, 45.0-46.7), UNC Lineberger Comprehensive Cancer Center had the highest incidence rate of female breast cancer (143.0; 95% CI, 141.7-144.3), and UT Health San Antonio MD Anderson Cancer Center had the highest incidence rate of cervical cancer (11.1; 95% CI, 10.5-11.8) (eTables 9 to 12 in Supplement 1).

All–cancer site mortality in the US was 148.4 per 100 000 population during 2017 to 2021 (95% CI, 148.2-148.6). Over the same period, catchment area all–cancer site mortality ranged from 115.2 (95% CI, 114.3-116.1) for the Tisch Cancer Institute to 181.1 (95% CI, 179.5-182.7) for the Markey Cancer Center (Figure 2B and eTable 2 in Supplement 1).

Racial or ethnic minority group cancer mortality was highest in the catchment areas of Stephenson Cancer Center (170.1; 95% CI, 165.9-174.3), Alvin J. Siteman Cancer Center (170.0; 95% CI, 165.9-174.1), and UPMC Hillman Cancer Center (165.8; 95% CI, 159.8-171.9), and lowest in the catchment areas of Dartmouth Cancer Center (85.0; 95% CI, 76.7-94.0), UCI Health Chao Family Comprehensive Cancer Center (107.5; 95% CI,105.2-109.9), and NYU Langone’s Laura and Isaac Perlmutter Cancer Center (108.5; 95% CI, 107.2-109.7) (eTables 3 to 7 in Supplement 1). Catchment area rural population cancer mortality rates were highest for Markey Cancer Center (199.5; 95% CI, 197.0-202.1), UF Health Cancer Center (199.0; 95% CI, 193.5-204.6), and Stephenson Cancer Center (190.8; 95% CI, 187.8-193.8) (eTable 8 in Supplement 1). The Stephenson Cancer Center catchment area had the highest site-specific mortality rates for female breast cancer (22.8; 95% CI, 22.0-23.7), colorectal cancer (16.4; 95% CI, 15.8-16.9), and cervical cancer (3.8; 95% CI, 3.5-4.2), while Markey Cancer Center had the highest mortality rate for lung cancer (52.7; 95% CI, 51.9-53.6) (eTables 9 to 12 in Supplement 1).

### Catchment Area Funding

Sixty-three of the 64 cancer centers had P30 CCSG funding reported in FY23. CCSG funding total costs ranged between $2 191 000 for the University of Hawaii Cancer Center catchment area and $14 034 522 for the Memorial Sloan-Kettering catchment area. Accounting for differences in indirect costs, CCSG funding direct costs ranged between $1 400 000 for the University of Hawaii Cancer Center catchment area and $9 531 954 for the Dana-Farber/Harvard Cancer Center catchment area, with a median of $2 735 247 and IQR of $1 824 969-$3 912 691 (eTable 13 in Supplement 1). All remaining analyses restrict CCSG funding to direct costs only.

**Figure 3.**
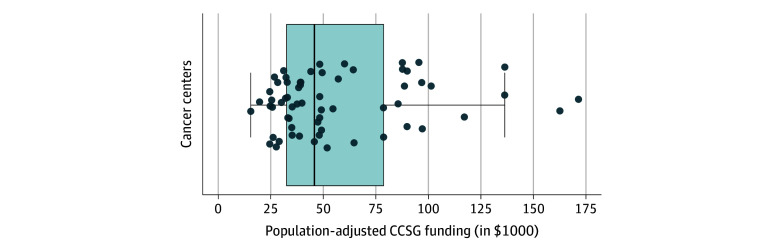
Distribution of Population-Adjusted Cancer Center Support Grant (CCSG) Funding for National Cancer Institute-Designated Cancer Centers During Fiscal Year 2023 CCSG funding includes awards for P30 Center Core Grants (opportunity numbers PAR-17-095, PAR-20-043, and/or PAR-21-321) and administrative supplements (opportunity number PA-20-272). Funding restricted to direct costs and adjusted per 100 000 people in the cancer center's catchment area. No CCSG funding was reported for Fox Chase Cancer Center in fiscal year 2023. Boxplot represents 25th percentile, 50th percentile, and 75th percentile. Whiskers extend to 1.5 times the IQR. Dots indicate individual cancer centers.

CCSG funding for cancer centers in FY23 was significantly associated with catchment area population size (*r* = 0.570; 95% CI, 0.375-0.716) and the total number of cancer cases (*r* = 0.620; 95% CI, 0.436-0.734), and cancer deaths (*r* = 0.631; 95% CI, 0.454-0.760). To better compare funding across cancer centers with substantially different catchment area population sizes, we considered CCSG funding per 100 000 people in the catchment area population. Adjusted according to population, cancer center funding ranged from $15 497 per 100 000 people for the City of Hope Comprehensive Cancer Center catchment area to $171 451 for the Montefiore Einstein Comprehensive Cancer Center catchment area (Figure 3). The median (IQR) population-adjusted CCSG funding among cancer centers was $45 931 ($32 543-$78 674).

**Figure 4.**
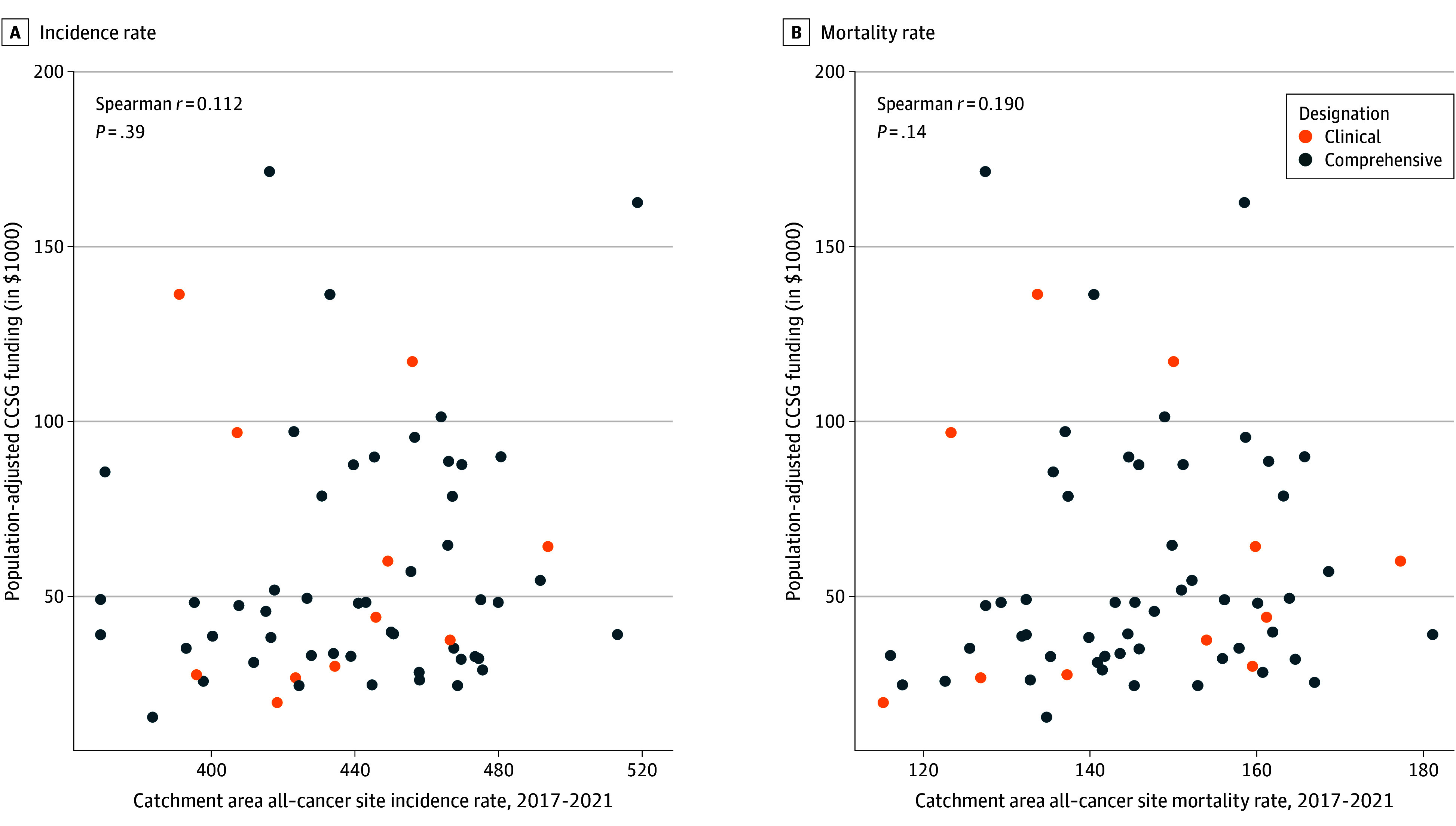
Association Between Catchment Area All–Cancer Site Incidence and Mortality Rates and Population-Adjusted Funding (A) Catchment area all–cancer site incidence vs Cancer Center Support Grant (CCSG) funding in 2023 per 100 000 people in catchment area population (n = 61), (B) catchment area all–cancer site mortality vs CCSG funding in 2023 per 100 000 people in catchment area population (n = 63). Catchment area incidence rates were not calculated for Indiana University Melvin and Bren Simon Comprehensive Cancer Center and University of Chicago Comprehensive Cancer Center due to 2017 to 2021 county-level cancer incidence rates in Indiana not meeting USCS publication criteria. No CCSG funding was reported for Fox Chase Cancer Center during fiscal year 2023. Association measured using Spearman rank correlation to account for lack of linearity.

Population-adjusted CCSG funding was negatively associated with catchment area minority racial or ethnic population percentage (r = −0.354; 95% CI, −0.553 to −0.116) and positively associated with rural population percentage (r = 0.356; 95% CI, 0.119 to 0.555) and percentage of persons living with a disability (r = 0.378; 95% CI, 0.143 to 0.572). There was no association between population-adjusted funding and catchment area population living below poverty. With respect to cancer burden, neither catchment area all–cancer site incidence nor mortality rates were associated with population-adjusted CCSG funding (Figure 4).

## Discussion

The catchment areas of NCI-designated cancer centers showed a great diversity in size and composition across the US. A great diversity in cancer incidence and mortality rates was also observed, although certain centers emerged as having higher rates across multiple cancer sites and population groups. CCSG funding for cancer centers also varied widely. Catchment areas with larger rural populations and percentages of people with a disability were associated with higher levels of population-adjusted CCSG funding. In contrast, funding was lower among cancer centers with larger percentages of their catchment area population belonging to a racial or ethnic minority group. No association was observed between CCSG funding amounts and catchment area cancer incidence or mortality rates, suggesting that currently there is no relationship between catchment area cancer burden and cancer centers funding. Overall, this analysis highlights which cancer centers face the largest cancer-related needs across their catchment area populations as well as potential areas of disconnect between current CCSG funding and the desire for cancer centers to impact the cancer burden and cancer health disparities that exist within their catchment area.

Previous research has described the catchment areas of NCI-designated cancer centers; however, this is the first study to calculate catchment area cancer incidence or to examine associations between cancer burden indicators and cancer center CCSG funding.^6,7^ These additions provide novel insights on need and resource allocation in light of the requirement that cancer centers perform some research and outreach focused on improving cancer outcomes within specific geographic boundaries.

Whether, and to what extent, a cancer center’s catchment area should impact its CCSG funding is an unexplored question. Prior to 2013, CCSG scoring was determined based on the national and global scientific impact of the cancer center’s research, added value of the center to the institution, and six essential characteristics of NCI-designated cancer centers. Since then consideration of catchment area-focused research and outreach has also been factored in, particularly through the creation of the Community Outreach and Engagement (COE) component in 2017.^3,4,5^ This component is responsible for various catchment area-related tasks and is broadly evaluated based upon “the scope, quality, and impact of the center’s community outreach and engagement activities on the burden of cancer in the Center’s stated catchment area.”^18^ NCI has articulated additional funding level criteria in recent years, including initial amounts for newly designated centers, tying funding increases to CCSG priority scores (a numerical assessment of the center’s overall impact given during CCSG review), overall cancer research funding, and specific opportunities for increasing funds for COE and other components.

Though various parts of a cancer center are expected to engage in efforts with catchment area relevance, COE is the one most directly tied to catchment areas and their inclusion in the CCSG. In fact, a 2021 study by Alaniz and Rebbeck^19^ found a strong association between the scoring of a cancer center’s COE component and the center’s overall CCSG priority score. Thus, these findings suggest a direct link between responsiveness to catchment area needs and cancer center funding. However, this relationship is potentially problematic given concerns that have been expressed about the inconsistency of COE scoring and assignment of some COE reviewers without an appropriate background in community-engaged research.^20^

Moreover, one could ask whether the amount of cancer need in a catchment area (eg, higher cancer incidence and mortality rates and larger proportions of vulnerable populations) should play a role in the amount of support a cancer center receives. Our findings of no and negative associations between population-adjusted funding and several of these metrics suggest this is not currently the case, despite the CCSG funding opportunity stating that “a long-term commitment to community outreach and engagement is required in order to have a profound impact on the cancer burden of a center’s catchment area.”^18^ As a result, some cancer centers are left with a paradox of combating substantial cancer-related needs in their catchment area with fewer financial and personnel resources, while other resource-rich centers could benefit from higher levels of existing support despite lower catchment area cancer burdens.

### Limitations

We acknowledge certain limitations with this study. First, although this cross-sectional study provides a comprehensive assessment of the current cancer burden of almost every NCI-designated cancer center, it does not account for progress made toward reducing catchment area cancer burden over time. However, longitudinal analysis would be complicated by the short amount of time since the introduction of the catchment area requirement and fluctuations in catchment area definitions over the first decade of this mandate. Second, additional sources of cancer center funding beyond the CCSG are not included due to lack of available data. Institutions are required to supplement CCSG funding amounts with other financial commitments to their work (eg, philanthropy, state/local government funding), and, in fact, this additional institutional support is a component that cancer centers are evaluated on. Nevertheless, CCSG funding is important and relevant to consider because it reflects the NCI’s commitment to the work of designated centers. Third, given the lack of publicly available data and funding level criteria, the current analysis did not include additional factors NCI may consider in determining the amount of funding that specific centers receive in their CCSG awards, such as the number of years since designation, historical CCSG funding levels, institutional affiliations, or cancer center size (eg, number of faculty members, size of existing total research funding). The purpose of this study was not to discern the complex process NCI uses to make funding level decisions, but only to provide descriptive and bivariate analyses appropriate for exploring patterns and stimulating discussion regarding the needs and funding of NCI-designated cancer centers.

## Conclusions

In this cross-sectional study of NCI-designated cancer centers, we found substantial differences in catchment area cancer burden—differences that were not reflected in the allocations of CCSG funding. In addition to the existing criteria, these findings suggest that the NCI could consider ways to include the degree of catchment area needs, such as cancer incidence and mortality rates and amounts of vulnerable populations, as part of the review criteria that drives priority scores and funding levels. This could be an effective strategy toward accomplishing the NCI’s stated objectives of reducing cancer burden and helping all people live longer, healthier lives.

## Notes

Supplement 1. **eTable 1.** Catchment area demographics **eTable 2.** All–cancer site incidence and mortality rates by catchment area **eTable 3. **Top and bottom 5 catchment area racial and ethnic minority group cancer incidence and mortality rates **eTable 4.** Top and bottom 5 catchment area Hispanic ethnicity incidence and mortality rates. **eTable 5.** Top and bottom 5 catchment area non-Hispanic American Indian/Alaska Native race and ethnicity cancer incidence and mortality rates **eTable 6.** Top and bottom 5 catchment area non-Hispanic Asian or Pacific Islander race and ethnicity cancer incidence and mortality rates **eTable 7.** Top and bottom 5 catchment area non-Hispanic Black race and ethnicity cancer incidence and mortality rates **eTable 8.** Top and bottom 5 catchment area rural population cancer incidence and mortality rates **eTable 9.** Top and bottom 5 catchment area female breast cancer incidence and mortality rates **eTable 10.** Top and bottom 5 catchment area lung and bronchus cancer incidence and mortality rates **eTable 11.** Top and bottom 5 catchment area colon and rectum cancer incidence and mortality rates **eTable 12.** Top and bottom 5 catchment area cervical cancer incidence and mortality rates **eTable 13. **Cancer Center Support Grant (CCSG) funding for National Cancer Institute-Designated Cancer Centers during fiscal year 2023 **eFigure. **Comparisons between 1-year cause-specific survival (CSS) rates among patients diagnosed with cancer in 2019 and 2020 by cancer site

Supplement 2.
Data Sharing Statement

## References

[1] The national cancer act of 1971. J Natl Cancer Inst. 1972;48(3):577-584. 5058965

[2] NCI-Designated Cancer Centers. National Cancer Institute. Accessed June 26, 2025. https://www.cancer.gov/research/infrastructure/cancer-centers

[3] Tai CG, Hiatt RA. The population burden of cancer: research driven by the catchment area of a cancer center. Epidemiol Rev. 2017;39(1):108-122. doi:10.1093/epirev/mxx001 28472310

[4] Paskett ED, Hiatt RA. Catchment areas and community outreach and engagement: the new mandate for NCI-designated cancer centers. Cancer Epidemiol Biomarkers Prev. 2018;27(5):517-519. doi:10.1158/1055-9965.EPI-17-1050 29716925

[5] Hiatt RA, Kobetz EN, Paskett ED. Catchment areas, community outreach and engagement revisited: the 2021 guidelines for cancer center support grants from the National Cancer Institute. Cancer Prev Res (Phila). 2022;15(6):349-354. doi:10.1158/1940-6207.CAPR-22-0034 35652232

[6] DelNero PF, Buller ID, Jones RR, . A national map of NCI-designated cancer center catchment areas on the 50th anniversary of the cancer centers program. Cancer Epidemiol Biomarkers Prev. 2022;31(5):965-971. doi:10.1158/1055-9965.EPI-21-1230 35101903 PMC9074106

[7] Leader AE, McNair C, Yurick C, . Assessing the coverage of US cancer center primary catchment areas. Cancer Epidemiol Biomarkers Prev. 2022;31(5):955-964. doi:10.1158/1055-9965.EPI-21-1097 35064067 PMC9081121

[8] Catchment areas of NCI-designated cancer centers. National Cancer Institute. Accessed June 26, 2025. https://gis.cancer.gov/ncicatchment/

[9] American Community Survey 5-Year Data (2019-2023). US Census Bureau. Accessed December 11, 2024. https://www.census.gov/programs-surveys/acs

[10] U.S. Department of Agriculture, Economic Research Service: Rural-Urban Continuum Codes, 2020. Accessed July 9, 2025. https://www.ers.usda.gov/data-products/rural-urban-continuum-codes

[11] How disability data are collected from the American Community Survey. US Census Bureau. Accessed May 9, 2025. https://www.census.gov/topics/health/disability/guidance/data-collection-acs.html

[12] United States Department of Health and Human Services, Centers for Disease Control and Prevention: National Program of Cancer Registries SEER*Stat Database: USCS Incidence Analytic Database with single ages—1998-2021—linked to county attributes.

[13] Siu AL; US Preventive Services Task Force. Screening for breast cancer: US Preventive Services Task Force recommendation statement. Ann Intern Med. 2016;164(4):279-296. doi:10.7326/M15-2886 26757170

[14] Moyer VA; US Preventive Services Task Force. Screening for lung cancer: US Preventive Services Task Force recommendation statement. Ann Intern Med. 2014;160(5):330-338. doi:10.7326/M13-2771 24378917

[15] U.S. Preventive Services Task Force. Screening for colorectal cancer: US Preventive Services Task Force recommendation statement. Ann Intern Med. 2008;149(9):627-637. doi:10.7326/0003-4819-149-9-200811040-00243 18838716

[16] Curry SJ, Krist AH, Owens DK, ; US Preventive Services Task Force. Screening for cervical cancer: US Preventive Services Task Force recommendation statement. JAMA. 2018;320(7):674-686. doi:10.1001/jama.2018.10897 30140884

[17] NIH Reporter. National Institutes of Health. Accessed June 27, 2025. https://reporter.nih.gov/

[18] Cancer Center Support Grants (CCSGs) for NCI-designated cancer centers. National Institutes of Health. Accessed June 27, 2025. https://grants.nih.gov/grants/guide/pa-files/PAR-21-321.html

[19] Alaniz M, Rebbeck TR. The role of community outreach and engagement in evaluation of NCI Cancer Center support grants. Cancer Causes Control. 2024;35(1):73-75. doi:10.1007/s10552-023-01770-3 37563423

[20] Thompson HS, Ashing KT, Barrett NJ, . The state of cancer-focused community outreach and engagement (COE): reflections of Black COE directors. J Natl Cancer Inst. 2024;116(10):1549-1554. doi:10.1093/jnci/djae138 38876978 PMC12116281

