# Errors in Figure 1 and Figure 4B

**DOI:** 10.1001/jamanetworkopen.2025.35685

**Published:** 2025-09-03

In the Original Investigation titled “Differences in the Cancer Burden and Current Funding of NCI-Designated Cancer Centers,”^1^ published August 1, 2025, the island of Puerto Rico was errantly shaded as a catchment area and should not have been included in Figure 1. Additionally, the x-axis of Figure 4B was labeled as the incidence rate and it should have been labeled as the mortality rate. This article has been corrected.^1^
