# Supplementary material for: Differences in the Cancer Burden and Current Funding of NCI-Designated Cancer Centers
Source: JAMA Netw Open. 2025 Aug 1;8(8):e2524564. doi: 10.1001/jamanetworkopen.2025.24564 (PMC12317354; doi:10.1001/jamanetworkopen.2025.24564)
Supplement: Supplement 1. — eTable 1. Catchment area demographics eTable 2. All–cancer site incidence and mortality rates by catchment area eTable 3. Top and bottom 5 catchment area racial and ethnic minority group cancer incidence and mortality rates eTable 4. Top and bottom 5 catchment area Hispanic ethnicity incidence and mortality rates. eTable 5. Top and bottom 5 catchment area non-Hispanic American Indian/Alaska Native race and ethnicity cancer incidence and mortality rates eTable 6. Top and bottom 5 catchment area non-Hispanic Asian or Pacific Islander race and ethnicity cancer incidence and mortality rates eTable 7. Top and bottom 5 catchment area non-Hispanic Black race and ethnicity cancer incidence and mortality rates eTable 8. Top and bottom 5 catchment area rural population cancer incidence and mortality rates eTable 9. Top and bottom 5 catchment area female breast cancer incidence and mortality rates eTable 10. Top and bottom 5 catchment area lung and bronchus cancer incidence and mortality rates eTable 11. Top and bottom 5 catchment area colon and rectum cancer incidence and mortality rates eTable 12. Top and bottom 5 catchment area cervical cancer incidence and mortality rates eTable 13. Cancer Center Support Grant (CCSG) funding for National Cancer Institute-Designated Cancer Centers during fiscal year 2023 eFigure. Comparisons between 1-year cause-specific survival (CSS) rates among patients diagnosed with cancer in 2019 and 2020 by cancer site [file jamanetwopen-e2524564-s001.pdf]

Supplemental Online Content

Burus T, McAfee CR, Hull PC. Differences in the cancer burden and current funding of NCI-designated cancer centers. *JAMA Netw Open*. 2025;8(8):e2524564. doi:10.1001/jamanetworkopen.2025.24564

eTable 1. Catchment area demographics

eTable 2. All cancer site incidence and mortality rates by catchment area

eTable 3. Top and bottom 5 catchment area racial and ethnic minority group cancer incidence and mortality rates

eTable 4. Top and bottom 5 catchment area Hispanic ethnicity incidence and mortality rates.

eTable 5. Top and bottom 5 catchment area non-Hispanic American Indian/Alaska Native race and ethnicity cancer incidence and mortality rates

eTable 6. Top and bottom 5 catchment area non-Hispanic Asian or Pacific Islander race and ethnicity cancer incidence and mortality rates

eTable 7. Top and bottom 5 catchment area non-Hispanic Black race and ethnicity cancer incidence and mortality rates

eTable 8. Top and bottom 5 catchment area rural population cancer incidence and mortality rates

eTable 9. Top and bottom 5 catchment area female breast cancer incidence and mortality rates

eTable 10. Top and bottom 5 catchment area lung and bronchus cancer incidence and mortality rates

eTable 11. Top and bottom 5 catchment area colon and rectum cancer incidence and mortality rates

eTable 12. Top and bottom 5 catchment area cervical cancer incidence and mortality rates

eTable 13. Cancer Center Support Grant (CCSG) funding for National Cancer Institute-Designated Cancer Centers during fiscal year 2023

eFigure. Comparisons between 1-year cause-specific survival (CSS) rates among patients diagnosed with cancer in 2019 and 2020 by cancer site

This supplemental material has been provided by the authors to give readers additional information about their work.

© 2025 Burus T et al. *JAMA Netw Open*.

eTable 1. Catchment area demographics, US Cancer Bureau American Community Survey 5-year Estimates, 2019-2023.[1] Racial and ethnic minority groups were defined according to the Census Bureau as Hispanic (any race), non-Hispanic American Indian or Alaska Native, non-Hispanic Asian or Pacific Islander, non-Hispanic Black, and other non-Hispanic race. Non-Hispanic Asian or Pacific Islander included non-Hispanic Asian alone and non-Hispanic Native Hawaiian or other Pacific Islander alone variables. Other non-Hispanic races included unknown non-Hispanic race and two or more non-Hispanic races. Rural population was calculated by totaling catchment area populations in counties with a Rural Urban Continuum Codes 2013 code of 4 through 9.

Cancer Center	Total Population (n)	Minority Race or Ethnicity (%)	Rural (%)	Living Below Poverty (%)	Living with a Disability (%)
Abramson Cancer Center (Philadelphia, PA)	7376967	40.2	0	11.6	13
Alvin J. Siteman Cancer Center (St. Louis, MO)	4792207	23.2	26.1	12.2	14.9
Atrium Health Wake Forest Baptist Comprehensive Cancer Center (Winston-Salem, NC)	5007470	38.3	9.3	12.6	12.6
Barbara Ann Karmanos Cancer (Detroit, MI)	6771479	29.7	10.7	13.4	14.8
Case Comprehensive Cancer Center (Cleveland, OH)	3975944	25.7	5.7	13.6	14.6
Chao Family Comprehensive Cancer (Orange, CA)	3164063	62.3	0	9.5	9.3
City of Hope Comprehensive Cancer Center (Duarte, CA)	17650194	71.6	0	12.5	10.9
Dan L. Duncan Comprehensive Cancer Center (Houston, TX)	7246731	66.4	0	13.6	10.3
Dana-Farber/Harvard Cancer Center (Boston, MA)	6992395	32.2	1.5	10	12.1
Dartmouth Cancer Center at Dartmouth Health (Lebanon, NH)	2033088	11.5	45.9	8.1	13.5
Duke Cancer Institute at Duke University Medical Center (Durham, NC)	11387110	42.3	17.6	13.3	13.2
Fox Chase Cancer Center (Philadelphia, PA)	7547050	39.2	0	11.8	13.2
Fred & Pamela Buffett Cancer Center (Omaha, NE)	1965926	23.8	34.4	10.3	12.1
Fred Hutchinson Cancer Center (Seattle, WA)	7740984	35.7	10.2	9.9	13.3
Harold C. Simmons Comprehensive Cancer Center (Dallas, TX)	7881263	56.7	0.9	10.5	10.1

© 2025 Burus T et al. *JAMA Netw Open*.

Helen Diller Family Comprehensive Cancer Center (San Francisco, CA)	13513178	63.9	1.5	11	11.3
Herbert Irving Comprehensive Cancer Center (New York, NY)	10806743	64.2	0	15.6	11.2
Holden Comprehensive Cancer Center (Iowa City, Iowa)	3195937	16.9	38.4	11	12.4
Hollings Cancer Center (Charleston, SC)	5212774	37.8	14	14.2	14.4
Huntsman Cancer Institute (Salt Lake City, UT)	10050316	31.6	20.2	10.7	12.6
Indiana University Melvin & Bren Simon Comprehensive Cancer Center (Indianapolis, IN)	6811752	23.9	21.8	12.2	13.8
Jonsson Comprehensive Cancer Center (Los Angeles, CA)	9848406	74.8	0	13.6	10.9
Laura & Isaac Perlmutter Cancer Center at NYU Langone Health (New York, NY)	10010770	57.3	0	12.7	10.4
Lineberger Comprehensive Cancer Center (Chapel Hill, NC)	10584340	39.4	20.9	13.2	13.4
Lombardi Comprehensive Cancer Center (Washington, DC)	6444529	61.8	0	9.5	8.9
Masonic Cancer Center (Minneapolis, MN)	5713716	23.3	22.1	9.2	11.4
Massey Comprehensive Cancer Center (Richmond, VA)	4135742	44.6	14.3	11	13.6
Mayo Clinic Cancer Center (Rochester, MN)	10453862	34.8	11.4	10.7	12.6
Mays Cancer Center at UT Health San Antonio MD Anderson Cancer Center (San Antonio, TX)	5116866	76.3	8.8	18.4	14.1
Memorial Sloan-Kettering Cancer Center (New York, NY)	29919552	49.9	2.3	12.3	11.6
Moffitt Cancer Center (Tampa, FL)	10484555	41.6	1.1	12	14.2
Montefiore Einstein Comprehensive Cancer Center (Bronx, NY)	1419250	91.2	0	26.9	16.5
Moores Comprehensive Cancer Center (La Jolla, CA)	3462101	58.6	0	10.8	10.9
Norris Comprehensive Cancer Center (Los Angeles, CA)	9848406	74.8	0	13.6	10.9
O'Neal Comprehensive Cancer Center (Birmingham, AL)	5054253	36.2	22.1	15.6	16.2
Oregon Health Science University Knight Cancer Institute (Portland, OR)	4238714	27.7	15.2	11.9	15.1
Robert H. Lurie Comprehensive Cancer Center (Chicago, IL)	8645679	51	0	10.9	10.5
Roswell Park Comprehensive Cancer Center (Buffalo, NY)	1548829	22.1	22.5	14.1	14.6
Rutgers Cancer Institute of New Jersey (New Brunswick, NJ)	9267014	48.1	0	9.8	10.6
Sidney Kimmel Cancer Center at Jefferson Health (Philadelphia, PA)	4958617	42.6	0	12.5	13.6
Sidney Kimmel Comprehensive Cancer Center (Baltimore, MD)	6170738	52.6	4.4	9.3	11.4
Stanford Cancer Institute (Stanford, CA)	7854978	68.1	0	9.4	10.3
Stephenson Cancer Center (Oklahoma City, OK)	3995260	37.2	32.4	15.3	16.8
Sylvester Comprehensive Cancer Center (Miami, FL)	6220716	71.3	1.3	13.1	11
The Ohio State University Comprehensive Cancer Center (Columbus, OH)	11780046	23.5	18.1	13.2	14.2
Tisch Cancer Institute (New York, NY)	8516202	68.7	0	17.4	11.7
University of Arizona Cancer Center (Tucson, AZ)	1880518	50.7	2.6	14	15.4
University of California at Davis Comprehensive Cancer Center (Sacramento, CA)	5184110	56.4	6	12.5	12.9

© 2025 Burus T et al. *JAMA Netw Open*.

University of Chicago Comprehensive Cancer Center (Chicago, IL)	8022400	53	0	11.6	10.8
University of Colorado Cancer Center (Aurora, CO)	5810774	34.3	12.3	9.4	11.2
University of Florida Health Cancer Center (Gainesville, FL)	2377943	33.2	13.1	14.7	15.9
University of Hawai'i Cancer Center (Honolulu, HI)	1445635	78.9	19.2	10	12.5
University of Kansas Cancer Center (Kansas City, KS)	4588871	26.9	22	11.9	13.6
University of Kentucky Markey Cancer Center (Lexington, KY)	4510725	17.7	38.1	16.1	17.7
University of Maryland Marlene & Stewart Greenebaum Comprehensive Cancer Center (Baltimore, MD)	5405712	56.4	0	9.2	11
University of Michigan Rogel Cancer Center (Ann Arbor, MI)	10051595	27	16.6	13.1	14.2
University of New Mexico Comprehensive Cancer Center (Albuquerque, NM)	2114768	63.5	33	18.1	16.7
University of Texas MD Anderson Cancer Center (Houston, TX)	29640343	60.1	9.8	13.8	12
University of Virginia Comprehensive Cancer Center (Charlottesville, VA)	3257582	22.2	30.2	13.6	16
University of Wisconsin Carbone Cancer Center (Madison, WI)	5892023	20.8	25.5	10.6	12
UPMC Hillman Cancer Center (Pittsburgh, PA)	3948792	14.2	20.8	11.9	15.3
Vanderbilt-Ingram Cancer Center (Nashville, TN)	8448577	27.7	22.9	13.9	15.3
Winship Cancer Institute (Atlanta, GA)	10822590	50.2	16.6	13.5	12.9
Yale Cancer Center (Hartford, CT)	3598348	37	5.8	10	12.1

© 2025 Burus T et al. *JAMA Netw Open*.

eTable 2. All cancer site incidence and mortality rates by catchment area, 2017-2021, US Cancer Statistics (USCS) Incidence Analytic Database and National Center for Health Statistics.[2,3] Incidence rates for Indiana University Melvin & Bren Simon Comprehensive Cancer Center and University of Chicago Comprehensive Cancer Center unavailable due to data from Indiana not meeting USCS standards.

**Cancer Center**	**Incidence**		**Mortality**	
	**Rate (95% CI)**	**Rank**	**Rate (95% CI)**	**Rank**
Abramson Cancer Center	469.8 (467.7 - 471.8)	12	151.2 (150.0 - 152.3)	25
Alvin J. Siteman Cancer Center	480.7 (478.1 - 483.2)	5	165.7 (164.3 - 167.2)	5
Atrium Health Wake Forest Baptist Comprehensive Cancer Center	474.5 (471.9 - 477.0)	9	155.9 (154.4 - 157.4)	20
Barbara Ann Karmanos Cancer Institute	457.9 (455.8 - 459.9)	22	160.7 (159.5 - 161.9)	12
Case Comprehensive Cancer Center	466.1 (463.4 - 468.8)	18	161.4 (159.9 - 163.0)	10
Chao Family Comprehensive Cancer Center	407.7 (404.7 - 410.7)	51	127.5 (125.8 - 129.1)	56
City of Hope Comprehensive Cancer Center	383.6 (382.4 - 384.9)	59	134.8 (134.1 - 135.5)	49
Dan L. Duncan Comprehensive Cancer Center	411.8 (409.6 - 414.0)	50	140.9 (139.6 - 142.3)	41
Dana-Farber/Harvard Cancer Center	433.0 (431.0 - 435.0)	38	140.5 (139.3 - 141.6)	42
Dartmouth Cancer Center at Dartmouth Health	464.0 (460.2 - 467.8)	20	149.0 (146.9 - 151.0)	29
Duke Cancer Institute at Duke University Medical Center	467.5 (465.9 - 469.2)	15	157.9 (156.9 - 158.9)	18
Fox Chase Cancer Center	473.3 (471.3 - 475.3)	11	151.7 (150.6 - 152.9)	24
Fred & Pamela Buffett Cancer Center	456.0 (452.0 - 460.0)	24	150.0 (147.8 - 152.3)	27
Fred Hutchinson Cancer Center	439.6 (437.6 - 441.6)	34	145.9 (144.7 - 147.0)	32
Harold C. Simmons Comprehensive Cancer Center	434.0 (431.8 - 436.2)	37	143.6 (142.3 - 144.9)	37
Helen Diller Family Comprehensive Cancer Center	400.3 (398.9 - 401.8)	53	131.8 (131.0 - 132.7)	54
Herbert Irving Comprehensive Cancer Center	427.9 (426.2 - 429.5)	40	116.1 (115.2 - 116.9)	63
Holden Comprehensive Cancer Center	491.6 (488.4 - 494.8)	4	152.2 (150.5 - 154.0)	23
Hollings Cancer Center at Medical University of South Carolina	434.4 (432.1 - 436.7)	36	159.5 (158.1 - 160.9)	15
Huntsman Cancer Institute	416.6 (414.8 - 418.4)	47	139.9 (138.8 - 140.9)	43

© 2025 Burus T et al. *JAMA Netw Open*.

Indiana University Melvin & Bren Simon Comprehensive Cancer Center	-	-	166.9 (165.7 - 168.2)	4
Jonsson Comprehensive Cancer Center	369.2 (367.5 - 370.8)	61	132.3 (131.4 - 133.3)	52
Laura & Isaac Perlmutter Cancer Center at NYU Langone Health	444.8 (443.1 - 446.5)	31	117.5 (116.6 - 118.4)	62
Lineberger Comprehensive Cancer Center	475.0 (473.3 - 476.8)	8	156.1 (155.1 - 157.1)	19
Lombardi Comprehensive Cancer Center	397.8 (395.7 - 399.9)	54	122.6 (121.5 - 123.8)	61
Masonic Cancer Center	479.9 (477.5 - 482.3)	6	143.0 (141.7 - 144.3)	38
Massey Comprehensive Cancer Center	445.8 (443.1 - 448.5)	29	161.2 (159.6 - 162.8)	11
Mayo Clinic Cancer Center	450.7 (449.1 - 452.4)	26	144.5 (143.6 - 145.5)	36
Mays Cancer Center at UT Health San Antonio MD Anderson Cancer Center	395.9 (393.4 - 398.4)	55	137.2 (135.8 - 138.7)	45
Memorial Sloan-Kettering Cancer Center	458.0 (457.0 - 458.9)	21	132.8 (132.3 - 133.3)	51
Moffitt Cancer Center	475.5 (473.9 - 477.2)	7	141.5 (140.6 - 142.3)	40
Montefiore Einstein Comprehensive Cancer Center	416.2 (411.6 - 420.9)	48	127.4 (124.9 - 130.0)	57
Moores Comprehensive Cancer Center	423.0 (420.0 - 426.0)	44	137.0 (135.3 - 138.7)	46
Norris Comprehensive Cancer Center	369.2 (367.5 - 370.8)	61	132.3 (131.4 - 133.3)	52
O'Neal Comprehensive Cancer Center	430.8 (428.4 - 433.1)	39	163.2 (161.8 - 164.6)	8
Oregon Health Science University Knight Cancer Institute	417.6 (415.1 - 420.1)	46	151.0 (149.5 - 152.5)	26
Robert H. Lurie Comprehensive Cancer Center	443.0 (441.2 - 444.9)	32	145.4 (144.3 - 146.5)	33
Roswell Park Comprehensive Cancer Center	518.7 (514.2 - 523.2)	1	158.5 (156.1 - 161.0)	17
Rutgers Cancer Institute of New Jersey	473.4 (471.6 - 475.3)	10	135.3 (134.3 - 136.2)	48
Sidney Kimmel Cancer Center at Jefferson Health	466.5 (464.0 - 469.0)	17	154.0 (152.6 - 155.4)	21
Sidney Kimmel Comprehensive Cancer Center	445.4 (443.2 - 447.6)	30	144.6 (143.4 - 145.9)	35
Stanford Cancer Institute	393.0 (391.1 - 394.9)	57	125.6 (124.5 - 126.6)	59
Stephenson Cancer Center	449.2 (446.4 - 452.0)	28	177.2 (175.5 - 179.0)	2
Sylvester Comprehensive Cancer Center	423.4 (421.4 - 425.5)	43	126.9 (125.8 - 128.0)	58
The Ohio State University Comprehensive Cancer Center	469.6 (468.0 - 471.2)	13	164.6 (163.7 - 165.6)	6
Tisch Cancer Institute at Icahn School of Medicine	418.3 (416.5 - 420.2)	45	115.2 (114.3 - 116.1)	64
UPMC Hillman Cancer Center	456.6 (454.0 - 459.3)	23	158.7 (157.2 - 160.1)	16
University of Arizona Cancer Center	391.1 (387.4 - 394.7)	58	133.7 (131.7 - 135.8)	50

© 2025 Burus T et al. *JAMA Netw Open*.

University of California at Davis Comprehensive Cancer Center	415.2 (412.8 - 417.6)	49	147.7 (146.3 - 149.1)	30
University of Chicago Comprehensive Cancer Center	-	-	145.9 (144.8 - 147.0)	31
University of Colorado Cancer Center	395.3 (393.0 - 397.5)	56	129.3 (128.0 - 130.6)	55
University of Florida Health Cancer Center	493.7 (490.2 - 497.3)	3	159.8 (157.9 - 161.7)	14
University of Hawai'i Cancer Center	407.2 (403.0 - 411.4)	52	123.3 (121.1 - 125.6)	60
University of Kansas Cancer Center	450.1 (447.5 - 452.7)	27	161.9 (159.4 - 164.5)	9
University of Kentucky Markey Cancer Center	513.1 (510.4 - 515.9)	2	181.1 (179.5 - 182.7)	1
University of Maryland Marlene & Stewart Greenebaum Comprehensive Cancer Center	438.9 (436.5 - 441.2)	35	141.8 (140.5 - 143.1)	39
University of Michigan Rogel Cancer Center	441.0 (439.3 - 442.6)	33	160.1 (159.1 - 161.1)	13
University of New Mexico Comprehensive Cancer Center	370.4 (367.0 - 373.8)	60	135.6 (133.6 - 137.6)	47
University of Texas MD Anderson Cancer Center	424.4 (423.3 - 425.5)	42	145.3 (144.7 - 145.9)	34
University of Virginia Comprehensive Cancer Center	426.6 (423.8 - 429.5)	41	163.9 (162.2 - 165.6)	7
University of Wisconsin Carbone Cancer Center	465.8 (463.6 - 468.1)	19	149.8 (148.6 - 151.1)	28
Vanderbilt-Ingram Cancer Center	455.6 (453.7 - 457.5)	25	168.6 (167.5 - 169.8)	3
Winship Cancer Institute	468.6 (466.8 - 470.4)	14	152.9 (151.9 - 154.0)	22
Yale Cancer Center	467.1 (464.3 - 470.0)	16	137.4 (135.9 - 138.9)	44

Abbreviation: CI = Confidence interval.

© 2025 Burus T et al. *JAMA Netw Open*.

eTable 3. Top and bottom 5 catchment area racial and ethnic minority group cancer incidence and mortality rates, 2017-2021.[2,3] Incidence rates for Indiana University Melvin & Bren Simon Comprehensive Cancer Center and University of Chicago Comprehensive Cancer Center unavailable due to data from Indiana not meeting USCS standards.

**Cancer Center**	**Rate (95% CI)**
**Incidence**	
**Top 5**	
Stephenson Cancer Center	499.6 (492.9 - 506.5)
Roswell Park Comprehensive Cancer Center	494.8 (482.2 - 507.7)
University of Wisconsin Carbone Cancer Center	487.8 (479.9 - 495.8)
UPMC Hillman Cancer Center	475.5 (465.7 - 485.6)
University of Kentucky Markey Cancer Center	472.5 (463.9 - 481.2)
**Bottom 5**	
University of Arizona Cancer Center	348.3 (342.3 - 354.5)
Chao Family Comprehensive Cancer Center	347.1 (343.0 - 351.2)
Stanford Cancer Institute	343.1 (340.6 - 345.5)
City of Hope Comprehensive Cancer Center	339.9 (338.3 - 341.4)
Jonsson Comprehensive Cancer Center	331.6 (329.6 - 333.5)
Norris Comprehensive Cancer Center	331.6 (329.6 - 333.5)
**Mortality**	
**Top 5**	
Stephenson Cancer Center	170.1 (165.9 - 174.3)
Alvin J. Siteman Cancer Center	170.0 (165.9 - 174.1)
UPMC Hillman Cancer Center	165.8 (159.8 - 171.9)
Case Comprehensive Cancer Center	165.3 (161.4 - 169.2)
Hollings Cancer Center at Medical University of South Carolina	165.1 (162.3 - 168.0)
**Bottom 5**	
Rutgers Cancer Institute of New Jersey	109.9 (108.4 - 111.5)
Stanford Cancer Institute	109.2 (107.8 - 110.7)
Laura & Isaac Perlmutter Cancer Center at NYU Langone Health	108.5 (107.2 - 109.7)
Chao Family Comprehensive Cancer Center	107.5 (105.2 - 109.9)
Dartmouth Cancer Center	85.0 (76.7 - 94.0)

Abbreviation: CI = Confidence interval.

© 2025 Burus T et al. *JAMA Netw Open*.

eTable 4. Top and bottom 5 catchment area Hispanic ethnicity incidence and mortality rates, 2017-2021.[2,3] Incidence rates for Indiana University Melvin & Bren Simon Comprehensive Cancer Center and University of Chicago Comprehensive Cancer Center unavailable due to data from Indiana not meeting USCS standards.

Cancer Center	Rate (95% CI)
**Incidence**	
**Top 5**	
University of Hawai'i Cancer Center	562.4 (535.4 - 590.3)
Roswell Park Comprehensive Cancer Center	443.2 (415.4 - 472.1)
Yale Cancer Center	424.8 (415.5 - 434.4)
Masonic Cancer Center	397.3 (381.2 - 413.8)
Sylvester Comprehensive Cancer Center	388.9 (385.8 - 391.9)
**Bottom 5**	
Massey Comprehensive Cancer Center	282.6 (269.1 - 296.6)
Sidney Kimmel Comprehensive Cancer Center	281.6 (273.5 - 289.9)
University of Virginia Comprehensive Cancer Center	267.4 (251.6 - 283.8)
UPMC Hillman Cancer Center	246.4 (226.6 - 267.3)
O'Neal Comprehensive Cancer Center	224.9 (212.1 - 238.1)
**Mortality**	
**Top 5**	
University of Hawai'i Cancer Center	185.4 (168.8 - 202.9)
University of New Mexico Comprehensive Cancer Center	132.3 (129.0 - 135.6)
University of Colorado Cancer Center	129.7 (125.9 - 133.7)
Mays Cancer Center at UT Health San Antonio MD Anderson Cancer Center	127.0 (125.2 - 128.9)
Barbara Ann Karmanos Cancer Institute	123.3 (115.8 - 131.1)
**Bottom 5**	
Dartmouth Cancer Center	70.4 (58.0 - 84.5)
University of Kentucky Markey Cancer Center	68.5 (59.9 - 77.8)
Alvin J. Siteman Cancer Center	68.4 (60.0 - 77.5)
University of Virginia Comprehensive Cancer Center	67.8 (59.5 - 76.8)
O'Neal Comprehensive Cancer Center	58.0 (51.2 - 65.3)

Abbreviation: CI = Confidence interval.

© 2025 Burus T et al. *JAMA Netw Open*.

eTable 5. Top and bottom 5 catchment area non-Hispanic American Indian/Alaska Native race and ethnicity cancer incidence and mortality rates, 2017-2021.[2,3] Incidence rates for Indiana University Melvin & Bren Simon Comprehensive Cancer Center and University of Chicago Comprehensive Cancer Center unavailable due to data from Indiana not meeting USCS standards.

**Cancer Center**	**Rate (95% CI)**
**Incidence**	
**Top 5**	
University of Hawai'i Cancer Center	978.4 (829.5 - 1146.8)
Stephenson Cancer Center	639.5 (626.9 - 652.3)
Masonic Cancer Center	629.5 (598.3 - 661.9)
University of Wisconsin Carbone Cancer Center	595.7 (564.9 - 627.7)
Fred Hutchinson Cancer Center	545.2 (524.8 - 566.1)
**Bottom 5**	
Vanderbilt-Ingram Cancer Center	131.6 (114.1 - 151.2)
Montefiore Einstein Comprehensive Cancer Center	128.1 (86.9 - 181.8)
Laura & Isaac Perlmutter Cancer Center at NYU Langone Health	126.3 (108.4 - 146.3)
O'Neal Comprehensive Cancer Center	110.8 (95.3 - 128.1)
Tisch Cancer Institute at Icahn School of Medicine	109.9 (93.0 - 129.0)
**Mortality**	
**Top 5**	
Masonic Cancer Center	211.1 (192.2 - 231.2)
Stephenson Cancer Center	196.8 (189.6 - 204.2)
University of Wisconsin Carbone Cancer Center	176.3 (158.9 - 195.0)
Holden Comprehensive Cancer Center	168.2 (129.5 - 214.3)
Fred & Pamela Buffett Cancer Center	164.4 (130.7 - 203.5)
**Bottom 5**	
Winship Cancer Institute at Emory University	37.2 (28.0 - 48.5)
Mays Cancer Center at UT Health San Antonio MD Anderson Cancer Center	35.6 (23.8 - 51.9)
Laura & Isaac Perlmutter Cancer Center at NYU Langone Health	23.1 (16.1 - 32.3)
Herbert Irving Comprehensive Cancer Center	20.2 (13.9 - 28.5)
Tisch Cancer Institute at Icahn School of Medicine	14.4 (8.9 - 22.1)

Abbreviation: CI = Confidence interval.

© 2025 Burus T et al. *JAMA Netw Open*.

eTable 6. Top and bottom 5 catchment area non-Hispanic Asian or Pacific Islander race and ethnicity cancer incidence and mortality rates, 2017-2021.[2,3] Incidence rates for Indiana University Melvin & Bren Simon Comprehensive Cancer Center and University of Chicago Comprehensive Cancer Center unavailable due to data from Indiana not meeting USCS standards.

Cancer Center	Rate (95% CI)
**Incidence**	
**Top 5**	
University of Hawai'i Cancer Center	364.9 (360.1 - 369.8)
Tisch Cancer Institute at Icahn School of Medicine	344.8 (340.5 - 349.1)
Laura & Isaac Perlmutter Cancer Center at NYU Langone Health	344.4 (340.3 - 348.5)
Herbert Irving Comprehensive Cancer Center	336.5 (332.6 - 340.4)
Montefiore Einstein Comprehensive Cancer Center	330.3 (310.4 - 351.1)
**Bottom 5**	
Hollings Cancer Center at Medical University of South Carolina	233.9 (219.4 - 249.1)
University of Virginia Comprehensive Cancer Center	230.6 (212.5 - 249.8)
Sylvester Comprehensive Cancer Center	228.1 (218.6 - 238.0)
Mays Cancer Center at UT Health San Antonio MD Anderson Cancer Center	207.1 (194.0 - 220.8)
Dartmouth Cancer Center	205.8 (186.1 - 226.9)
**Mortality**	
**Top 5**	
Masonic Cancer Center	116.1 (108.5 - 124.0)
Holden Comprehensive Cancer Center	116.0 (101.5 - 131.8)
Stephenson Cancer Center	114.7 (103.3 - 126.9)
University of Hawai'i Cancer Center	114.1 (111.5 - 116.7)
Oregon Health Science University Knight Cancer Institute	109.7 (103.0 - 116.7)
**Bottom 5**	
Dartmouth Cancer Center at Dartmouth Health	74.7 (62.2 - 88.8)
University of Michigan Rogel Cancer Center	74.7 (70.0 - 79.6)
Barbara Ann Karmanos Cancer Institute	74.6 (69.2 - 80.3)
Rutgers Cancer Institute of New Jersey	72.4 (69.9 - 75.0)
Yale Cancer Center	67.0 (60.5 - 73.8)

Abbreviation: CI = Confidence interval.

© 2025 Burus T et al. *JAMA Netw Open*.

eTable 7. Top and bottom 5 catchment area non-Hispanic Black race and ethnicity cancer incidence and mortality rates, 2017-2021.[2,3] Incidence rates for Indiana University Melvin & Bren Simon Comprehensive Cancer Center and University of Chicago Comprehensive Cancer Center unavailable due to data from Indiana not meeting USCS standards.

**Cancer Center**	**Rate (95% CI)**
**Incidence**	
**Top 5**	
University of Wisconsin Carbone Cancer Center	560.2 (547.7 - 572.9)
Holden Comprehensive Cancer Center	557.4 (531.9 - 583.8)
Roswell Park Comprehensive Cancer Center	515.4 (499.0 - 532.1)
University of Kentucky Markey Cancer Center	495.9 (485.4 - 506.7)
UPMC Hillman Cancer Center	485.9 (473.8 - 498.2)
**Bottom 5**	
Chao Family Comprehensive Cancer Center	370.2 (347.4 - 394.1)
University of Arizona Cancer Center	350.0 (329.5 - 371.3)
University of Hawai'i Cancer Center	347.6 (313.0 - 384.7)
Dartmouth Cancer Center	331.1 (294.5 - 370.7)
University of New Mexico Comprehensive Cancer Center	331.0 (307.4 - 356.0)
**Mortality**	
**Top 5**	
University of Wisconsin Carbone Cancer Center	218.5 (210.3 - 227.0)
Holden Comprehensive Cancer Center	205.2 (188.6 - 222.6)
Alvin J. Siteman Cancer Center	199.3 (194.3 - 204.5)
UPMC Hillman Cancer Center	195.9 (188.1 - 203.8)
Stephenson Cancer Center	195.9 (188.1 - 203.8)
**Bottom 5**	
University of Hawai'i Cancer Center	134.4 (110.9 - 160.8)
Tisch Cancer Institute at Icahn School of Medicine	132.6 (130.4 - 134.7)
Herbert Irving Comprehensive Cancer Center	132.0 (130.0 - 134.0)
Laura & Isaac Perlmutter Cancer Center at NYU Langone Health	130.2 (128.1 - 132.5)
Dartmouth Cancer Center	122.2 (98.0 - 149.9)

Abbreviation: CI = Confidence interval.

© 2025 Burus T et al. *JAMA Netw Open*.

eTable 8. Top and bottom 5 catchment area rural population cancer incidence and mortality rates, 2017-2021.[2,3] Eighteen cancer centers have no rural population. Incidence rates for Indiana University Melvin & Bren Simon Comprehensive Cancer Center and University of Chicago Comprehensive Cancer Center unavailable due to data from Indiana not meeting USCS standards.

**Cancer Center**	**Rate (95% CI)**
**Incidence**	
**Top 5**	
University of Kentucky Markey Cancer Center	522.4 (518.2 - 526.6)
University of Florida Health Cancer Center	514.8 (505.6 - 524.3)
Roswell Park Comprehensive Cancer Center	504.3 (495.0 - 513.7)
Alvin J. Siteman Cancer Center	497.6 (492.5 - 502.6)
Holden Comprehensive Cancer Center	491.6 (486.7 - 496.4)
**Bottom 5**	
University of Hawai'i Cancer Center	401.3 (391.9 - 410.9)
Sylvester Comprehensive Cancer Center	384.8 (369.2 - 401.1)
University of Colorado Cancer Center	354.6 (349.0 - 360.2)
University of New Mexico Comprehensive Cancer Center	343.2 (337.5 - 348.9)
University of Arizona Cancer Center	317.9 (297.3 - 339.6)
**Mortality**	
**Top 5**	
University of Kentucky Markey Cancer Center	199.5 (197.0 - 202.1)
University of Florida Health Cancer Center	199.0 (193.5 - 204.6)
Stephenson Cancer Center	190.8 (187.8 - 193.8)
Vanderbilt-Ingram Cancer Center	186.3 (184.0 - 188.5)
Alvin J. Siteman Cancer Center	184.6 (181.6 - 187.6)
**Bottom 5**	
University of Hawai'i Cancer Center	136.3 (131.1 - 141.8)
Dana-Farber/Harvard Cancer Center	134.7 (126.5 - 143.3)
Sylvester Comprehensive Cancer Center	127.8 (119.2 - 137.0)
University of Colorado Cancer Center	125.5 (122.2 - 128.8)
University of Arizona Cancer Center	114.1 (102.1 - 127.1)

Abbreviation: CI = Confidence interval.

© 2025 Burus T et al. *JAMA Netw Open*.

eTable 9. Top and bottom 5 catchment area female breast cancer incidence and mortality rates, 2017-2021.[2,3] Incidence rates for Indiana University Melvin & Bren Simon Comprehensive Cancer Center and University of Chicago Comprehensive Cancer Center unavailable due to data from Indiana not meeting USCS standards.

**Cancer Center**	**Rate (95% CI)**
**Incidence**	
**Top 5**	
Lineberger Comprehensive Cancer Center	143.0 (141.7 - 144.3)
Yale Cancer Center	143.0 (140.7 - 145.2)
Duke Cancer Institute at Duke University Medical Center	141.9 (140.6 - 143.2)
Atrium Health Wake Forest Baptist Comprehensive Cancer Center	140.7 (138.7 - 142.6)
Masonic Cancer Center	140.4 (138.6 - 142.3)
**Bottom 5**	
Sylvester Comprehensive Cancer Center	119.8 (118.2 - 121.3)
University of New Mexico Comprehensive Cancer Center	116.3 (113.5 - 119.0)
University of Arizona Cancer Center	114.6 (111.7 - 117.5)
Montefiore Einstein Comprehensive Cancer Center	112.7 (109.4 - 116.0)
Mays Cancer Center at UT Health San Antonio MD Anderson Cancer Center	111.5 (109.6 - 113.3)
**Mortality**	
**Top 5**	
Stephenson Cancer Center	22.8 (22.0 - 23.7)
Massey Comprehensive Cancer Center	22.7 (21.8 - 23.5)
Vanderbilt-Ingram Cancer Center	21.5 (20.9 - 22.0)
Hollings Cancer Center at Medical University of South Carolina	21.4 (20.7 - 22.1)
University of Kentucky Markey Cancer Center	21.4 (20.6 - 22.2)
Sidney Kimmel Cancer Center	21.4 (20.7 - 22.1)
**Bottom 5**	
Sylvester Comprehensive Cancer Center	17.2 (16.6 - 17.7)
Laura & Isaac Perlmutter Cancer Center at NYU Langone Health	17.0 (16.5 - 17.4)
University of Arizona Cancer Center	16.8 (15.8 - 17.9)
Dana-Farber/Harvard Cancer Center	16.0 (15.5 - 16.6)
University of Hawai'i Cancer Center	16.0 (14.8 - 17.2)

Abbreviation: CI = Confidence interval.

© 2025 Burus T et al. *JAMA Netw Open*.

eTable 10. Top and bottom 5 catchment area lung and bronchus cancer incidence and mortality rates, 2017-2021.[2,3] Incidence rates for Indiana University Melvin & Bren Simon Comprehensive Cancer Center and University of Chicago Comprehensive Cancer Center unavailable due to data from Indiana not meeting USCS standards.

Cancer Center	Rate (95% CI)
**Incidence**	
**Top 5**	
University of Kentucky Markey Cancer Center	84.4 (83.3 - 85.5)
Alvin J. Siteman Cancer Center	72.3 (71.3 - 73.2)
Vanderbilt-Ingram Cancer Center	68.0 (67.3 - 68.7)
Roswell Park Comprehensive Cancer Center	67.0 (65.5 - 68.6)
University of Florida Health Cancer Center	65.4 (64.2 - 66.6)
**Bottom 5**	
Stanford Cancer Institute at Stanford University	35.6 (35.0 - 36.2)
City of Hope Comprehensive Cancer Center	34.5 (34.1 - 34.9)
University of New Mexico Comprehensive Cancer Center	32.7 (31.8 - 33.7)
Jonsson Comprehensive Cancer Center	32.6 (32.1 - 33.1)
Norris Comprehensive Cancer Center	32.6 (32.1 - 33.1)
**Mortality**	
**Top 5**	
University of Kentucky Markey Cancer Center	52.7 (51.9 - 53.6)
Stephenson Cancer Center	46.2 (45.3 - 47.1)
Vanderbilt-Ingram Cancer Center	45.7 (45.1 - 46.3)
Alvin J. Siteman Cancer Center	44.4 (43.6 - 45.1)
Indiana University Melvin & Bren Simon Comprehensive Cancer Center	43.6 (43.0 - 44.3)
**Bottom 5**	
Jonsson Comprehensive Cancer Center	22.3 (21.9 - 22.7)
Norris Comprehensive Cancer Center	22.3 (21.9 - 22.7)
Lombardi Comprehensive Cancer Center	22.2 (21.7 - 22.7)
Laura & Isaac Perlmutter Cancer Center at NYU Langone Health	22.1 (21.7 - 22.5)
Herbert Irving Comprehensive Cancer Center	21.4 (21.1 - 21.8)
Tisch Cancer Institute at Icahn School of Medicine	20.9 (20.5 - 21.3)

Abbreviation: CI = Confidence interval.

© 2025 Burus T et al. *JAMA Netw Open*.

eTable 11. Top and bottom 5 catchment area colon and rectum cancer incidence and mortality rates, 2017-2021.[2,3] Incidence rates for Indiana University Melvin & Bren Simon Comprehensive Cancer Center and University of Chicago Comprehensive Cancer Center unavailable due to data from Indiana not meeting USCS standards.

Cancer Center	Rate (95% CI)
**Incidence**	
**Top 5**	
University of Kentucky Markey Cancer Center	45.9 (45.0 - 46.7)
Alvin J. Siteman Cancer Center	40.3 (39.6 - 41.1)
Stephenson Cancer Center	40.3 (39.4 - 41.1)
O'Neal Comprehensive Cancer Center	40.1 (39.4 - 40.8)
Holden Comprehensive Cancer Center	40.0 (39.1 - 40.9)
**Bottom 5**	
Stanford Cancer Institute	32.7 (32.2 - 33.3)
Oregon Health Science University Knight Cancer Institute	32.6 (31.8 - 33.3)
Chao Family Comprehensive Cancer Center	31.5 (30.7 - 32.4)
University of Colorado Cancer Center	31.3 (30.7 - 32.0)
Dana-Farber/Harvard Cancer Center	31.2 (30.6 - 31.7)
**Mortality**	
**Top 5**	
Stephenson Cancer Center	16.4 (15.8 - 16.9)
University of Kentucky Markey Cancer Center	16.3 (15.8 - 16.8)
University of Virginia Comprehensive Cancer Center	15.1 (14.6 - 15.6)
Indiana University Melvin & Bren Simon Comprehensive Cancer Center	14.8 (14.4 - 15.2)
Vanderbilt-Ingram Cancer Center	14.8 (14.4 - 15.1)
**Bottom 5**	
Tisch Cancer Institute at Icahn School of Medicine	10.9 (10.6 - 11.2)
Herbert Irving Comprehensive Cancer Center	10.8 (10.6 - 11.1)
Dana-Farber/Harvard Cancer Center	10.7 (10.4 - 11.0)
Chao Family Comprehensive Cancer Center	10.5 (10.1 - 11.0)
Yale Cancer Center	10.5 (10.0 - 10.9)

Abbreviation: CI = Confidence interval.

© 2025 Burus T et al. *JAMA Netw Open*.

eTable 12. Top and bottom 5 catchment area cervical cancer incidence and mortality rates, 2017-2021.[2,3] Incidence rates for Indiana University Melvin & Bren Simon Comprehensive Cancer Center and University of Chicago Comprehensive Cancer Center unavailable due to data from Indiana not meeting USCS standards.

Cancer Center	Rate (95% CI)
**Incidence**	
**Top 5**	
Mays Cancer Center at UT Health San Antonio MD Anderson Cancer Center	11.1 (10.5 - 11.8)
Stephenson Cancer Center	10.2 (9.6 - 10.9)
University of Kentucky Markey Cancer Center	9.7 (9.1 - 10.3)
University of Texas MD Anderson Cancer Center	9.6 (9.4 - 9.9)
University of Florida Health Cancer Center	9.6 (8.8 - 10.5)
**Bottom 5**	
University of Wisconsin Carbone Cancer Center	5.9 (5.4 - 6.3)
Masonic Cancer Center	5.4 (5.0 - 5.8)
Dartmouth Cancer Center	5.3 (4.7 - 6.0)
Yale Cancer Center	5.3 (4.8 - 5.8)
Dana-Farber/Harvard Cancer Center	4.7 (4.4 - 5.1)
**Mortality**	
**Top 5**	
Stephenson Cancer Center	3.8 (3.5 - 4.2)
Mays Cancer Center at UT Health San Antonio MD Anderson Cancer Center	3.7 (3.3 - 4.0)
O'Neal Comprehensive Cancer Center	3.2 (2.9 - 3.6)
University of Florida Health Cancer Center	3.2 (2.7 - 3.7)
University of Texas MD Anderson Cancer Center	2.9 (2.7 - 3.0)
**Bottom 5**	
Dartmouth Cancer Center	1.5 (1.2 - 1.8)
Yale Cancer Center	1.4 (1.2 - 1.6)
University of Wisconsin Carbone Cancer Center	1.4 (1.2 - 1.6)
Masonic Cancer Center	1.3 (1.1 - 1.5)
Dana-Farber/Harvard Cancer Center	1.1 (1.0 - 1.3)

Abbreviation: CI = Confidence interval.

© 2025 Burus T et al. *JAMA Netw Open*.

eTable 13. Cancer Center Support Grant (CCSG) funding for National Cancer Institute-Designated Cancer Centers during fiscal year 2023, NIH RePORTER.[4] CCSG funding includes awards for P30 Center Core Grants (opportunity numbers PAR-17-095, PAR-20-043, and/or PAR-21-321) and administrative supplements (opportunity number PA-20-272). Funding restricted to direct costs and adjusted per 100,000 people in the cancer center's catchment area. No CCSG funding was reported for Fox Chase Cancer Center in fiscal year 2023.

Cancer Center	Direct Costs	Population-Adjusted Direct Costs
Abramson Cancer Center (Philadelphia, PA)	$6,469,825	$87,703
Alvin J. Siteman Cancer Center (St. Louis, MO)	$4,310,055	$89,939
Atrium Health Wake Forest Baptist Comprehensive Cancer Center (Winston-Salem, NC)	$1,615,554	$32,263
Barbara Ann Karmanos Cancer (Detroit, MI)	$1,917,029	$28,310
Case Comprehensive Cancer Center (Cleveland, OH)	$3,523,721	$88,626
Chao Family Comprehensive Cancer (Orange, CA)	$1,499,999	$47,407
City of Hope Comprehensive Cancer Center (Duarte, CA)	$2,735,247	$15,497
Dan L. Duncan Comprehensive Cancer Center (Houston, TX)	$2,256,661	$31,140
Dana-Farber/Harvard Cancer Center (Boston, MA)	$9,531,954	$136,319
Dartmouth Cancer Center at Dartmouth Health (Lebanon, NH)	$2,060,264	$101,337
Duke Cancer Institute at Duke University Medical Center (Durham, NC)	$4,009,614	$35,212
Fox Chase Cancer Center (Philadelphia, PA)	-	-
Fred & Pamela Buffett Cancer Center (Omaha, NE)	$2,302,825	$117,137
Fred Hutchinson Cancer Center (Seattle, WA)	$6,783,814	$87,635
Harold C. Simmons Comprehensive Cancer Center (Dallas, TX)	$2,655,734	$33,697
Helen Diller Family Comprehensive Cancer Center (San Francisco, CA)	$5,224,036	$38,659
Herbert Irving Comprehensive Cancer Center (New York, NY)	$3,580,279	$33,130
Holden Comprehensive Cancer Center (Iowa City, Iowa)	$1,744,988	$54,600
Hollings Cancer Center (Charleston, SC)	$1,566,273	$30,047
Huntsman Cancer Institute (Salt Lake City, UT)	$3,846,939	$38,277
Indiana University Melvin & Bren Simon Comprehensive Cancer Center (Indianapolis, IN)	$1,732,858	$25,439
Jonsson Comprehensive Cancer Center (Los Angeles, CA)	$3,847,324	$39,065
Laura & Isaac Perlmutter Cancer Center at NYU Langone Health (New York, NY)	$2,475,671	$24,730

© 2025 Burus T et al. *JAMA Netw Open*.

Lineberger Comprehensive Cancer Center (Chapel Hill, NC)	$5,193,267	$49,066
Lombardi Comprehensive Cancer Center (Washington, DC)	$1,660,729	$25,770
Masonic Cancer Center (Minneapolis, MN)	$2,760,159	$48,308
Massey Comprehensive Cancer Center (Richmond, VA)	$1,822,126	$44,058
Mayo Clinic Cancer Center (Rochester, MN)	$4,107,381	$39,291
Mays Cancer Center at UT Health San Antonio MD Anderson Cancer Center (San Antonio, TX)	$1,414,000	$27,634
Memorial Sloan-Kettering Cancer Center (New York, NY)	$7,816,219	$26,124
Moffitt Cancer Center (Tampa, FL)	$3,040,955	$29,004
Montefiore Einstein Comprehensive Cancer Center (Bronx, NY)	$2,433,316	$171,451
Moores Comprehensive Cancer Center (La Jolla, CA)	$3,362,258	$97,116
Norris Comprehensive Cancer Center (Los Angeles, CA)	$4,839,225	$49,137
O'Neal Comprehensive Cancer Center (Birmingham, AL)	$3,978,058	$78,707
Oregon Health Science University Knight Cancer Institute (Portland, OR)	$2,197,263	$51,838
Robert H. Lurie Comprehensive Cancer Center (Chicago, IL)	$4,177,380	$48,318
Roswell Park Comprehensive Cancer Center (Buffalo, NY)	$2,518,331	$162,596
Rutgers Cancer Institute of New Jersey (New Brunswick, NJ)	$3,041,834	$32,824
Sidney Kimmel Cancer Center at Jefferson Health (Philadelphia, PA)	$1,861,386	$37,538
Sidney Kimmel Comprehensive Cancer Center (Baltimore, MD)	$5,545,069	$89,861
Stanford Cancer Institute (Stanford, CA)	$2,764,346	$35,192
Stephenson Cancer Center (Oklahoma City, OK)	$2,401,277	$60,103
Sylvester Comprehensive Cancer Center (Miami, FL)	$1,665,001	$26,765
The Ohio State University Comprehensive Cancer Center (Columbus, OH)	$3,775,148	$32,047
Tisch Cancer Institute (New York, NY)	$1,674,944	$19,668
University of Arizona Cancer Center (Tucson, AZ)	$3,770,792	$95,492
University of California at Davis Comprehensive Cancer Center (Sacramento, CA)	$2,564,565	$136,375
University of Chicago Comprehensive Cancer Center (Chicago, IL)	$2,371,334	$45,742
University of Colorado Cancer Center (Aurora, CO)	$2,807,612	$34,997
University of Florida Health Cancer Center (Gainesville, FL)	$2,805,729	$48,285
University of Hawai'i Cancer Center (Honolulu, HI)	$1,528,447	$64,276
University of Kansas Cancer Center (Kansas City, KS)	$1,400,000	$96,843
University of Kentucky Markey Cancer Center (Lexington, KY)	$1,764,499	$39,118
University of Maryland Marlene & Stewart Greenebaum Comprehensive Cancer Center (Baltimore, MD)	$1,827,812	$39,831
University of Michigan Rogel Cancer Center (Ann Arbor, MI)	$1,778,509	$32,901

© 2025 Burus T et al. *JAMA Netw Open*.

University of New Mexico Comprehensive Cancer Center (Albuquerque, NM)	$4,833,194	$48,084
University of Texas MD Anderson Cancer Center (Houston, TX)	$1,810,154	$85,596
University of Virginia Comprehensive Cancer Center (Charlottesville, VA)	$7,266,801	$24,517
University of Wisconsin Carbone Cancer Center (Madison, WI)	$1,611,583	$49,472
UPMC Hillman Cancer Center (Pittsburgh, PA)	$3,808,801	$64,643
Vanderbilt-Ingram Cancer Center (Nashville, TN)	$4,826,633	$57,130
Winship Cancer Institute (Atlanta, GA)	$2,654,390	$24,526
Yale Cancer Center (Hartford, CT)	$2,829,767	$78,641

© 2025 Burus T et al. *JAMA Netw Open*.

eFigure 1. Association between catchment area demographics and population-adjusted Cancer Center Support Grant (CCSG) funding.[1,4] (A) Catchment area racial and ethnicity minority group population, (B) catchment area rural population, (C) catchment area population living below poverty, (D) catchment area population living with a disability. Population-adjusted CCSG funding is the total direct costs of funding under CCSG opportunity numbers standardized per 100,000 persons in the cancer centers catchment area. Association between demographics and funding assessed using the Spearman rank correlation coefficient.

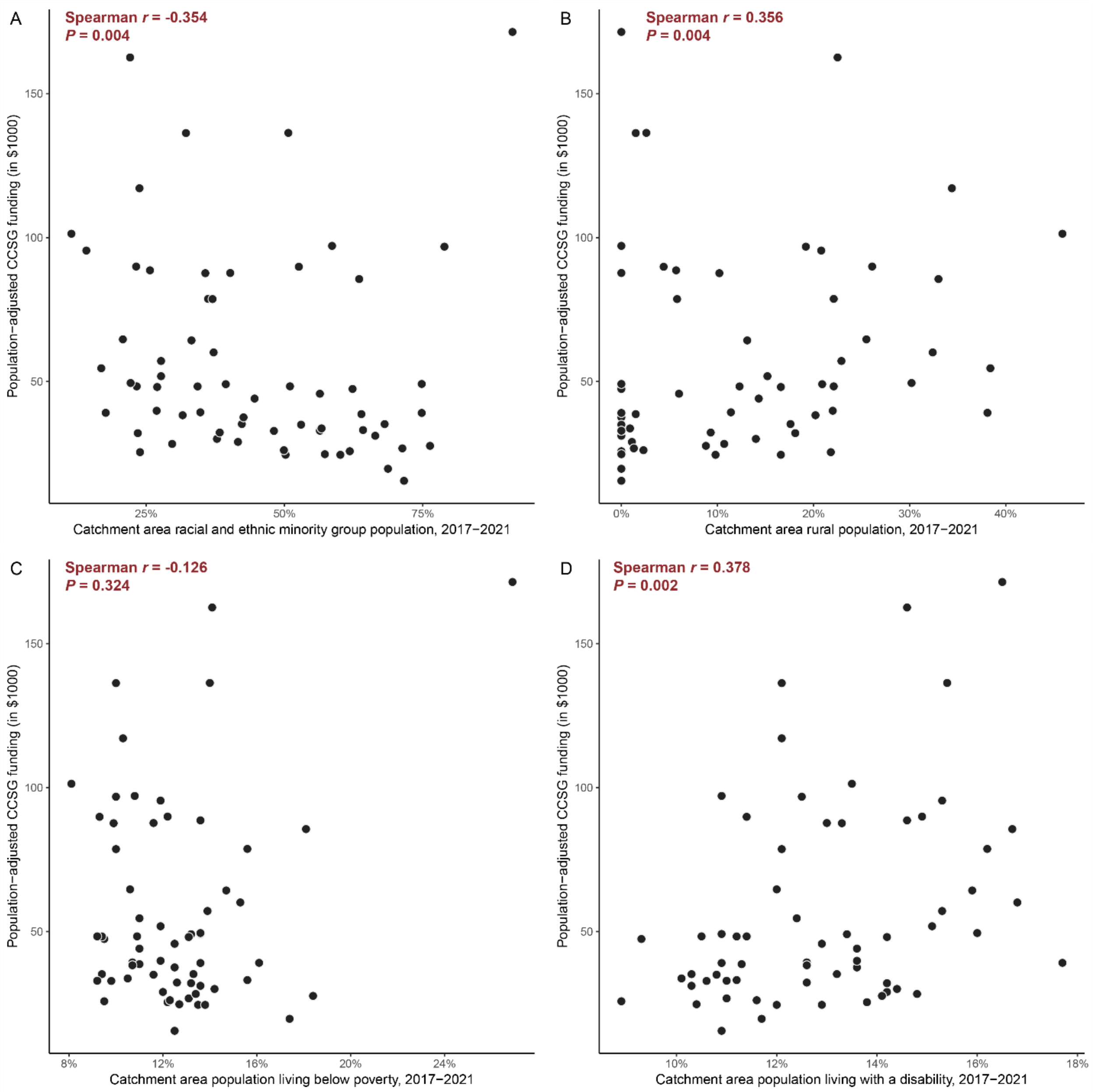

© 2025 Burus T et al. *JAMA Netw Open*.

References

1. U.S. Census Bureau. American Community Survey 5-Year Data (2019-2023). Published online 2024. Accessed December 11, 2024. https://www.census.gov/programs-surveys/acs

2. Centers for Disease Control and Prevention, National Center for Health Statistics. National Vital Statistics System, Mortality 1999-2020 on CDC WONDER Online Database, released in 2021. Data are from the Multiple Cause of Death Files, 1999-2020, as compiled from data provided by the 57 vital statistics jurisdictions through the Vital Statistics Cooperative Program. Accessed July 7, 2024. http://wonder.cdc.gov/ucd-icd10.html

3. National Cancer Institute, DCCPS, Surveillance Research Program. Surveillance, Epidemiology, and End Results (SEER) Program (www.seer.cancer.gov) SEER*Stat Database: Mortality - All COD, Aggregated With County, Total U.S. (1990-2021) <Katrina/Rita Population Adjustment> - Linked To County Attributes - Total U.S., 1969-2022 Counties. Published online May 2024.

4. National Institutes of Health. NIH RePORTER. August 18, 2024. Accessed August 24, 2024. https://reporter.nih.gov/

© 2025 Burus T et al. *JAMA Netw Open*.
