# Supplementary material for: Differences in the Cancer Burden and Current Funding of NCI-Designated Cancer Centers
Source: JAMA Netw Open. 2025 Aug 1;8(8):e2524564. doi: 10.1001/jamanetworkopen.2025.24564 (PMC12317354; doi:10.1001/jamanetworkopen.2025.24564)
Supplement: Supplement 2. — Data Sharing Statement [file jamanetwopen-e2524564-s002.pdf]

Data Sharing Statement

Data
Data available: Yes
Data types: Data (not involving human participants), Data dictionary
How to access data: Data on catchment area cancer incidence and mortality used in this study has been made available by the authors on their website https://cancerinfocus.org. All other data used are publicly available through the indicated sources.
When available: With publication

Supporting Documents
Document types: None

Additional Information
Who can access the data: General public
Types of analyses: Any
Mechanisms of data availability: Data will be general available to all persons under any circumstances
Any additional restrictions: n/a
